# Characterization the mitochondrial genome of *Acrossocheilus Labiatus* (Cypriniformes, Acrossocheilus) and its phylogeny

**DOI:** 10.1080/23802359.2019.1636723

**Published:** 2019-07-12

**Authors:** Yongguang Xie, Dunxue Chen, Yandong Niu, Xiaodong Wang, Huahui Luo, Xianjun Zhou

**Affiliations:** a College of Animal Science, Guizhou University, Guiyang, P.R. China;; b Hunan Academy of Forestry, Changsha, P.R. China

**Keywords:** *Acrossocheilus labiatus*, mitochondrial genome, gene arrangement

## Abstract

The first complete mitochondrial genome of *Acrossocheilus Labiatus* from the Qingshui River were reported in this study with accession number MG878098. The overall nucleotide composition was 31.13% A, 25.10% T, 27.52% C, 16.24% G, respectively. Phylogenetic analysis shows that the *A. parallens* and *A. Hemispinus* showed a closest phylogenetic relationship, then clustal with *A. Labiatus.*

*Acrossocheilus* is commonly known as monitoring and evaluators of the environmental contaminants by its sensitive to aquatic pollution (Liu et al. 2016). In the natural state, *Acrossocheilus* is widely distributed in the the gravel sediment of rivers and streams. There are more than 10 species belong to *Acrossocheilus* were reported (Xie et al. 2016). Unfortunately, as one of the most important species in *Acrossocheilus*, *Acrossocheilus labiatus* is relatively less studied.

For the purpose above, the *A. labiatus* was obtained from the Qingshui River (N107.87, E26.54) and soaked in ethyl alcohol (95%). The total genomic DNA was extracted from skeletal muscle tissues of the fishes and then stored at −80 °C until use in Guizhou University. Then, 14 pairs of primers were used to amplify contiguous, overlapping segments of the complete mitochondrial genome of *A. Labiatus.* The complete mitochondrial genome of *A. Labiatus* has been deposited in the GenBank with accession number MG878098 (16,586 bp in length). The gene arrangement and transcriptional direction were similar to those of the typical teleosts mitogenomes (Chen et al. 2012), which included 13 protein-coding genes, 2 ribosomal RNA (rRNA) genes, 22 transfer RNA (tRNA) genes and a displacement loop locus (D-loop). As observed in other fish species, the G contents were very low (16.24%) (Yu et al. 2016). The others were 31.13%A, 25.10%T, 27.52%C, respectively.

To confirm the phylogenetic placement of *A. labiatus* in the *Acrossocheilus,* 11 Acrossocheilus fishes and *Cyprinus carpio* (out-groups) were chosen. Phylogenetic analyses were constructed by the neighbor-joining (NJ) method performed on both amino acid levels and total mitogenomes (Zou et al. 2017) (Figure 1).

**Figure 1.**
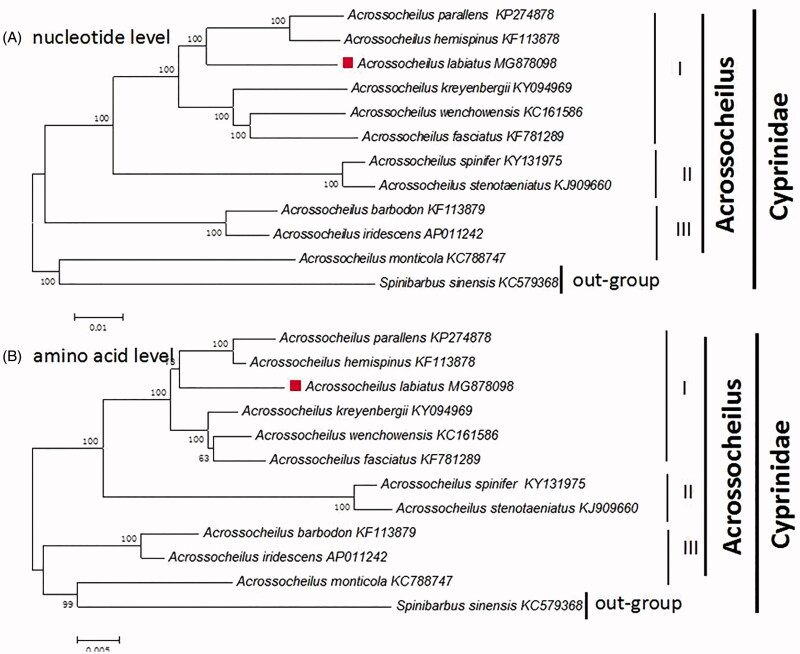
The phylogenetic analyses investigated using N–J analysis indicated evolutionary relationships among 11 *Acrossocheilus* based on the nucleotide **(**A) and amino acid (B). *Cyprinus carpio* (GenBank:KU159761) was used as the outgroup.

In our study, the phylogenetic placement of *A. Labiatus* was strongly supported by both amino acid levels and total mitogenomes with the same result (Figure 1). The 11 *Acrossocheilus* were divided into three clades (I, II, and III). In group I, there were 6 *Acrossocheilus* fishes clustal together, while the group II with 2, group III with 2. However, the *A. Monticola* shows a closest phylogenetic relationship with *Cyprinus carpio*(out-group) which reveal the *Acrossocheilus* was not nonmonophyletic. Our work confirmed that the *A. Labiatus* shows a closest phylogenetic relationship with *A. parallens* and *A. Hemispinus* (Xie et al. 2016). The *A. parallens* and *A. Hemispinus* showed a closest phylogenetic relationship, then clustal with *A. Labiatus.* The result provided a powerful evidence that the *A. Labiatus* is a separate species which is in accordance with the previous studies.
